# Competition Among Mental Health Organizations: Environmental Drivers and Strategic Responses

**DOI:** 10.1007/s10488-020-01079-2

**Published:** 2020-09-12

**Authors:** Alicia C. Bunger, Mi Sun Choi, Hannah MacDowell, Thomas Gregoire

**Affiliations:** 1 College of Social Work, Ohio State University, 1947 College Road, Columbus, OH 43210 USA; 2 Present Address: Department of Social Welfare, Silla University, Busan, South Korea; 3 Community Naloxone Distribution Consultant, Ohio Department of Health, Columbus, Ohio USA

**Keywords:** Competition, Leadership, Mental health services, Policy

## Abstract

While mental health system reforms have sought to leverage competition in the private sector to improve service quality and costs, competition among mental health organizations is poorly understood. To inform future studies about the impact of policy and system reforms on mental health organizations and service delivery, this qualitative study explores (1) resources for which organizations compete most intensively, (2) drivers of competition, and (3) leaders’ strategic organizational responses. Semi-structured phone interviews were conducted with 15 organizational leaders (CEO’s, executive directors) representing about 22% of organizations in the regional mental health market. Interviews covered leaders’ perceptions about competition, and their strategic responses. Porter’s seminal framework on competition was used to interpret codes and themes. Intensive competition for personnel was driven by workforce shortages, new for-profit organizations, and alternative employment opportunities. In response, organizations have attended to wages/benefits, recruitment, and retention. However, strong community need, expanded insurance coverage, and a history of local strategic responses that created service niches appeared to have minimized competition for financial resources in the region. Competition for funding and clients was expected to intensify under systems reform, and in anticipation, organizations were expanding services. Leaders also feared for the viability of smaller organizations in highly competitive environments. Consistent with theory on competition, mental health organizations compete and respond in ways that might improve services. However, the goals of privatization may have been unrealized because of minimal competition for funding and clients, and intense competition may undermine quality.

Over the past several decades, mental health system reforms have shifted responsibility for delivering services from the public to private systems, comprised of local nonprofit and for-profit community-based organizations. Privatization introduces competition among mental health organizations, defined as rivalry for the same resources (e.g., public contracts) to deliver similar services (Barman 2002; Hunt 1997). By awarding contracts based on effectiveness and efficiency, funders (e.g., governmental agencies, insurance carriers, foundations) can replace providers who are underperforming (Johnston and Girth 2012). Competitive pressure can lead organizations to learn, develop competencies, and become efficient (Barnett et al. 1994; Eikenberry and Kluver 2004). Theoretically, funders and policy making bodies can leverage competition among organizations to improve service quality and impact while containing costs, consistent with New Public Management principles that underpin privatization reforms in health and mental health (Cuellar and Haas-Wilson 2009; Frank and Glied 2006; Smith and Smyth 1996; Van Slyke 2003).

Despite the purported benefits of a private system based on competition and a history of policy reforms that sought to in4crease competition, mental health systems still suffer from persistent service accessibility, coordination, quality, and affordability problems in the US and abroad (Bruns et al. 2016; Hogan 2003; Owens et al. 2002; Westra et al. 2016; Xu et al. 2019). Recent evidence suggests that competition among service organizations is generally weak (Domański 2012; Savas 2002); but strong competition does not necessarily lead to better performance (Brunjes 2019). In fact, within mental health and social service contexts, intensive competition can divert resources away from mission-oriented service delivery (toward securing funding and personnel), destabilize or undermine collaboration, and cause other negative consequences for service delivery (Berrone et al. 2016; Bunger 2013; Clark and Dorwart 1992; Milward and Provan 2000). These issues suggest that the full benefits of competition have not been realized, and policies intended to intensify competition could have potentially adverse effects on mental health and other human services (Berrone et al. 2016; Porter and Teisberg 2006).

Although these questions have been explored in healthcare (e.g. Gaynor et al. 2015), competition may cause mental health organizations to respond somewhat differently, and is not well-understood. Health and mental health are knowledge-intensive fields, where organizations compete based on service quality (Tuckman 1998). However, evaluating quality could be more challenging in mental health than traditional healthcare, given the complex, ambiguous, and often untested nature of community-based psychosocial treatment (Sandfort 2003). Instead of relying on clear outcome indicators, funders and clients may rely on more “opaque” or subjective quality signals when choosing mental health organizations such as reputation, or compliance with professional standards (von Nordenflycht 2010). Therefore competition, and how it is managed in mental health care might be highly responsive to shifting institutional standards and pressures (Tuckman 1998). The intensity of competition for key resources, environmental shifts that intensify competition, and how leaders strategically respond has received limited empirical attention. This gap limits our understanding of how and why introducing competitive market principles via system reforms, policies, and regulations may or may not impact mental health service quality and costs. Since organizational leaders are responsible for strategic decisions (Vangen and Huxham 2003), how competitive organizational environments are *perceived* by leaders may matter more than objective assessments of the environment for making strategic management decisions (Smircich and Stubbart 1985). Thus, this study addresses this gap and examined competition from the perspective of mental health organizational leaders. Drawing on Porter’s classic strategic management framework on competition, we explored (1) the resources for which organizations compete most intensively, (2) the drivers of competition, and (3) leaders’ strategic organizational responses. These results have implications for how leaders manage competition among mental health organizations, and also generate new insights about how mental health policy reforms that emphasize competition are perceived and enacted in the field.

## Do Mental Health Organizations Compete? For What?

Competition arises when multiple organizations rely on limited resources to produce similar services or products (Barman 2002; Hunt 1997)—for instance, when one organization successfully wins a new contract or grant, they reduce the resources available to others. Although sometimes considered a negative relationship, competition within a regional market can be healthy. With multiple provider options in a region, clients or funders can exercise choice. For instance, clients can choose which mental health organization they visit based on treatment match, affordability, and/or accessibility. Funders can choose which organizations to support based on innovativeness, performance, or efficiency. As a result, competition for resources can drive organizations to deliver efficient, high quality, and consumer-driven service delivery (Smith and Smyth 1996; Van Slyke 2003).

Generally, health and human service markets are regarded as weak with little direct competition for contracts (Lamothe 2014; Savas 2002). However, competition among mental health organizations extends beyond direct rivalry for government contracts—organizations also compete for private funding, insured or well-paying clients, qualified staff, and political and community support (Alexander et al. 2008; Bunger 2013; McBeath et al. 2012; Romzek et al. 2012). Organizations may compete for these resources with varying intensity. Little is known about the forms of competition that leaders encounter, which are felt most acutely, or require a response. Therefore, our first objective is to explore leaders’ perceptions about the intensity of competition for key organizational resources.

## What Drives Competition?

**Fig. 1:**
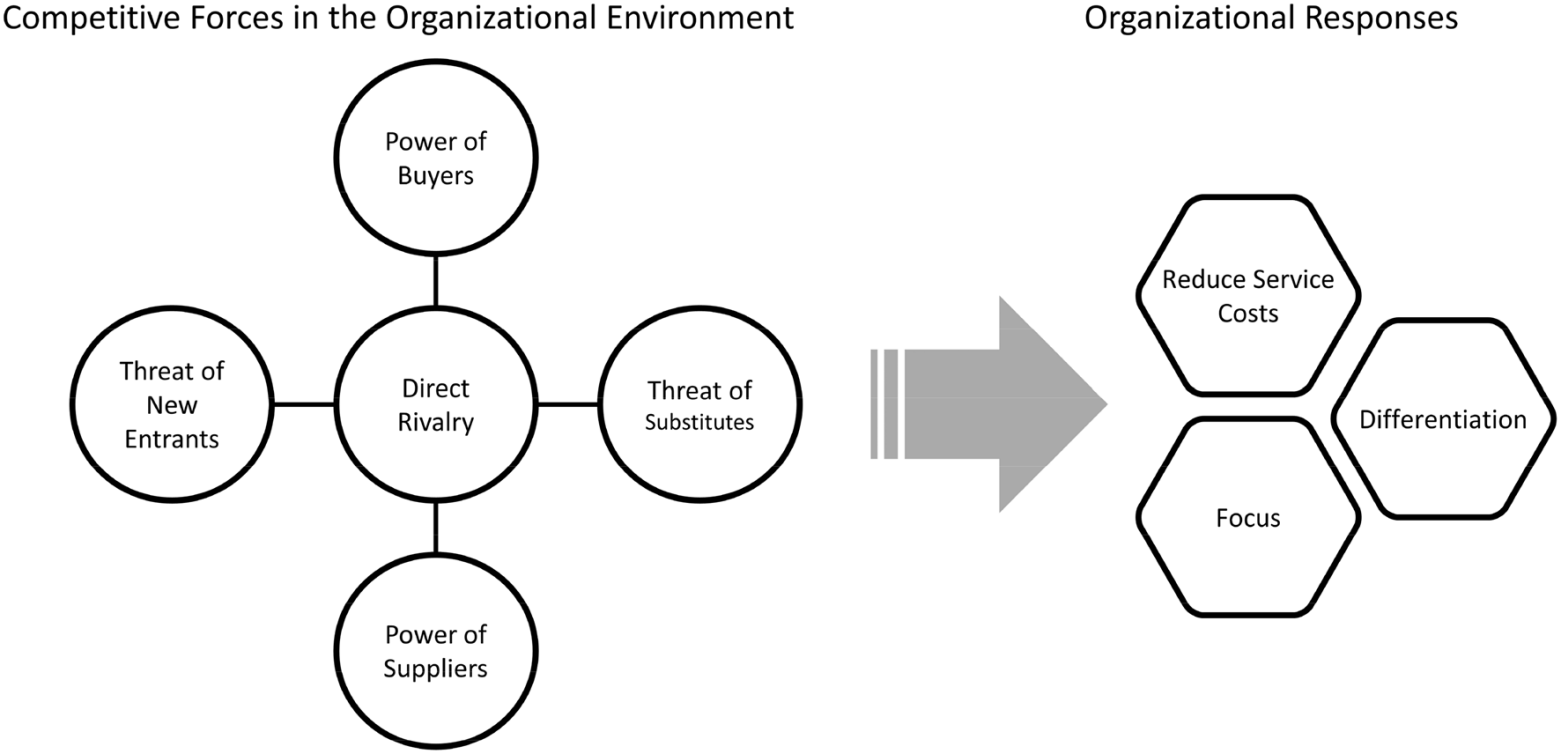
Porter’s five competitive forces and three responses

Porter’s (1980, 2008a, b) seminal work identified five forces in the external environment that can be leveraged through policy reforms to drive the intensity of competition and expected organizational responses (Fig. 1). The first three forces include other organizations that deliver mental health services (considered “horizontal” competitors). First, *direct rivals* (existing organizations that require similar types of resources) exert pressure on one another (Barman 2002; Hunt 1997). The greater the number of mental health organizations within a market, the more intensively organizations must compete for limited resources (Baum and Singh 1994; Gray and Schlesinger 2002). Second, *new organizations* that enter the market can intensify competition, as they draw on the same pool of funding, clients, staff, and other resources as existing organizations. In mental health care, new organizations may form to meet new community needs, although strict government regulations, accreditation requirements, and the substantial capital needed to establish a mental health organization restrict their ease of entry which may limit competition (e.g., Lethbridge 2011). Third, *substitutes for traditional mental health organizations* such as tele-health providers, e-health interventions, no-cost religious counseling, or other credible alternatives also heighten competition since they vie for similar funding and clients (Tuckman 1998). In the interest of consumer protections and quality, federal and state governments regulate mental health care by setting guidelines and standards for care. By imposing or removing these regulations that govern organizations, professionals, and service delivery, policy makers can influence the number of organizations or providers in a market.

The last two competitive forces emanate from “supplier” and “buyer” entities (considered “vertical” competition). The availability of *supplies* (a fourth force) can drive competition*.* Personnel are key “supplies” in mental health service delivery, whereby shortages of qualified and talented clinicians drive competition (e.g. Breedveld et al. 2006). Fifth, those who “buy” or purchase mental health services also influence competition. *Buyers* include the clients seeking mental health care—as the number of clients seeking services increases, organizations do not need to compete as intensively with one another as when there are few clients seeking services. Yet, in mental health care, services are often purchased directly by insurance providers or other funders (both public and private). These buyers are especially powerful since they purchase services for large groups of clients. As their resources remain stable or decline, organizations must compete more intensively for their contracts and grants (Bunger et al. 2017). Notably, purchasers are also instrumental in enforcing policy because funding for mental health care (grants, contracts to deliver fee-for-service, etc.) is often contingent upon compliance with professional standards, regulations, and other requirements. For instance, funding might be restricted to those with a particular type of accreditation, those that are only nonprofit, or those that deliver evidence-based practices. Therefore, purchasers influence the intensity of vertical competition and reinforce the regulations and policies that influence horizontal competition. As buyers and suppliers become more powerful, competition is likely to intensify.

In sum, competition among mental health organizations is likely driven by external pressures from other organizations, and powerful buyers and suppliers. However, the strength and salience of these competitive forces particularly in mental health are unclear which has implications for informing system reform efforts and policies. Our second objective is to identify external competitive pressures for mental health organizations.

## How Do Executive Leaders Respond to Competition?

Executive leaders respond to competition in three strategic ways (Barman 2002; Dess and Davis 1984; Porter 2008a) (Fig. 1). First, in response to strong competition, organizations may *reduce service costs* (Boehm 1996). For instance, to appeal to funders and consumers especially when they have strong bargaining power (and can choose organizations based on cost), organizations might find ways to deliver their services more affordably. Notably, streamlining services without sacrificing quality can be challenging given the extensive fixed costs (e.g. office space, IT systems, billing) involved in mental health service delivery, and limited organizational financial assets (Bunger et al. 2019). Therefore, reducing costs might be a rare response (Domański 2012). Second, mental health organizations may respond by *differentiating themselves* in terms of the types or quality of services they deliver (Barman 2002; Boehm 1996). Differentiation involves delivering a unique service, or developing a unique approach to obtaining resources/supplies that allows organizations to justify asking for a higher rate; for example, organizations develop new services or programs, improve service quality, or adopt new treatment innovations to distinguish themselves from their rivals (Compagni et al. 2014; Domański 2012; Proctor et al. 2007). Third, organizations respond by *focusing* on a narrower client population or geographic area (also called market segmentation) which can lead to service specializations, a strong consumer-orientation, and a stable supply of resources (Apenteng et al. 2015; McBeath, Jolles, Chuang et al. 2014).

The number of direct rivals, new organizations, and substitutes all generate strong competition for funding and clients (“buyers”) and personnel (“suppliers”). As buyers and suppliers become more powerful (e.g., their availability declines) they have more choice over which organization they fund, seek treatment from, or accept employment. These choices can theoretically drive costs and quality (Grønbjerg 1993; Tuckman 1998).

However, heightened competition especially in times of fiscal retrenchment can negatively impact mental health organizations. High-quality service delivery can suffer as intensive competition for financial resources can lead to difficulties meeting service demands (Reid and Brown 2008), or mission drift, where financial interests trump mission-driven work (Clark and Dorwart 1992). Intensive competition can also undermine collaboration especially without trust (Bunger 2013; Hu et al. 2019; Milward and Provan 2000). Mental health organizations experience strong pressures from funders, accreditors, and communities to partner (Guo and Acar 2005) with the same organizations with whom they compete for funding, staff, and other resources. Collaborating with a competitor (coined “coopetition”) (Brandenburger and Nalebuff 1996) is common in human services (Bunger et al. 2014; Valente et al. 2008) and linked to innovation and problem solving (e.g. Gnyawali and Park 2009). However, these complex relationships are risky because in a competitive environment, one partner’s success in winning a new contract or grant, hiring qualified personnel, or recruiting new clients could come at the expense of their partner, leading to relationship strain, failure, or dissolution (Baker et al. 1998). As competition intensifies, strong and meaningful forms of collaboration are uncommon (Bunger et al. 2017) which could suggest that intense competition compromises coordinated care delivery. Ultmately, intensive competition for scarce resources can even threaten the long term viability of organizations. Exactly how leaders respond to competition and whether their strategic responses improve coordination, availability, accessibility, quality, and effectiveness is also unclear. Therefore, the third objective of this study is to explore leaders’ response to competition.

## Methods

### Study Setting

This study examined competition among mental health organizations within the same regional market in central Ohio (Franklin and six contiguous counties: Delaware, Licking, Fairfield, Pickaway, Madison, and Union). This region is a major metropolitan area with over 2 million residents (about 17% of the state’s population) and is the home of the state capital (Columbus, Franklin County). The region is predominantly urban, and benefits from local allocations for behavioral health care through county tax levies (in addition to other sources) to support service delivery. This region was selected because, as an urban area, it is likely to have a fairly high density of service providers and thus a stronger competitive market compared to other suburban or rural areas (Girth et al. 2012); organizational leaders in competitive markets are likely more attuned to competition and able to reflect on their experiences and observations (Alexander et al. 2008). Many organizations were preparing for the most recent mental health system transformation—a transition in the state’s Medicaid program from a fee-for-service to a managed care model (representing a shift in the buying power of public funders) when the study was conducted in autumn/winter 2017. We narrowed in on a regional subset during a focused period of time to reduce geographic and industry-level variations, so we could more clearly identify regional policy, practice, and community influences on competition.

### Sampling

Participants included executive leaders (e.g. the executive director, chief executive officer, or other administrative leader) from 15 private mental health organizations. Leaders were predominantly female (60%), with executive experience from 1.5 to 40 years.

We used a purposive and multistage approach. Our sampling frame included 57 unique mental health organizations in the regional market listed in The National Directory of Mental Health Treatment Facilities (hosted by SAMHSA). This directory lists facilities licensed by state behavioral health authorities, and information gathered through the National Mental Health Services Survey (NMHSS). We used a random selection process to generate a representative sample of organizations since our goal was to explore leaders’ perceptions about competition (and were not exploring hypotheses about whether these perceptions might vary across different types of mental health organizations). To uncover themes iteratively over time, we conducted six staged rounds of recruitment, where we randomly selected up to five organizations in each round. We invited 24 leaders, and 15 agreed to participate (63%). After fifteen interviews, we reached saturation of themes where we did not uncover new insights or themes about competition in the region. To maximize efficiency and reduce data collection burden, we ended recruitment.

### Data Collection

**Fig. 2:**
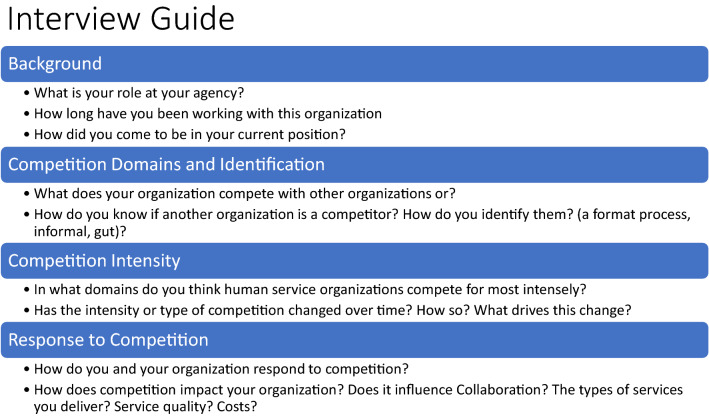
Topics Covered in Interview Guide

Semi-structured phone interviews were conducted with each leader that lasted 45–60 min, by up to two members of the research team. The interview guide asked about the different resources for which leaders competed with other organizations, the intensity and drivers of competition, and how leaders and their organizations have responded (see Fig. 2 for interview guide). Discussion topics were ordered in terms of complexity and abstraction (leading with more simple and concrete topics such as the types of resources that organizations compete for) (Schensul et al. 1999). All participants received a copy of the informed consent and interview guide in advance. Interviews were recorded (using UberConference platform), and professionally transcribed. The interviewers also took notes during interviews and prepared a reflection immediately after each interview to identify preliminary themes. To incentivize participation, leaders were offered a $30 gift card and a report of study findings. We also drew on publicly available information about the organizations as catalogued in SAMHSA’s behavioral health treatment locator to better understand the target populations served (e.g. children, adults), and type of facility (e.g. outpatient) among the organizations in the region and the sample. For the organizations in our sample, we also conducted searches on Guidestar.org and the internet and to assess for/non-profit status, and annual revenue. Using annual revenue as a proxy for size (Jaskyte 2013), we categorized organizations as small (less than $2 M), medium ($2–$10 M), and large (over $10 M). Study procedures were approved by the first author’s home Institutional Review Board (IRB).

### Analysis

Transcripts and reflections were analyzed using an iterative open-coding process consistent with a modified grounded theory approach (Strauss and Corbin 1998). First, two coders independently reviewed all interview reflections and transcripts to develop an initial codebook. Second, the codebook was refined iteratively; two coders applied the codebook to a transcript independently, compared and discussed codes (for three transcript iterations). Third, once coding consistency established, each transcript was coded using the Coding Analysis Toolkit (Shulman 2017). Codes and themes were further interpreted via memoing and through the lens of Porter’s (2008a, b) theory about distinctive competitive forces, and strategic responses. Quotes are presented with basic organizational characteristics (size, and for/nonprofit status) to illustrate themes and context; we made minimal edits to enhance readability.

## Results

The leaders interviewed represented 15 mental health organizations that described themselves (in SAMHSA’s behavioral health treatment locator) as outpatient (53%), residential (33%), community mental health (20%), and/or partial hospitalization (7%) treatment facilities (Table 1). Most served all age groups (73%); only 13% did not serve children, and 13% did not serve adults. Organizations represented were predominantly nonprofit (87%); these nonprofits generated a median annual revenue of $4.4 M (mean = $7 M, SD = $5.5 M) although ranged from $794,096 to $16,956,774. (Similar information about for-profit organizational revenue is not available publicly). Our analysis uncovered two major thematic streams about competition among regional mental health organizations. One stream emerged around intensive competition for qualified personnel, and a second focused on limited/stable competition for clients and financial resources (although this was anticipated to intensify with a statewide transition to a Medicaid managed care program). The nature, drivers, and responses to these different types of competition were distinct. Finally, leaders also described generally negative perceptions about the impact of competition on their organization and the larger system’s ability to meet community needs. Instead, many emphasized the importance of collaboration for remaining competitive.

**Table 1:** Organizational characteristics as recorded in SAMHSA behavioral health treatment locator

	Region (n = 57)		Sample (n = 15)	
	n	%	n	%
Facility type				
Outpatient	28	49.1%	8	53.3%
Community mental health	11	19.3%	3	20.0%
Psychiatric unit	7	12.3%	0	0.0%
Residential—child	4	7.0%	2	13.3%
Residential—adult	5	8.8%	2	13.3%
Residential—other	1	1.8%	1	6.7%
Partial hospitalization	4	7.0%	1	6.7%
Age group				
Children	42	73.7%	12	80.0%
Young adults	47	82.5%	13	86.7%
Adults	43	75.4%	12	80.0%
Seniors	38	66.7%	11	73.3%

Categories are not mutually exclusive

### Strong Competition for Personnel

Nearly all the leaders described intensive competition for personnel. Many agreed that competition for talented staff was far more intense than competition for other organizational resources including funding. In nearly every interview, directors described substantial difficulty filling open positions. As one leader of a large non-profit organization commented, “We will advertise for a position, and sometimes get one or two resumes for that position.”

Leaders reported that they compete with a wide range of other organizations within and outside of the mental health system for talent. Hospitals and other healthcare organizations were considered the strongest competitors because they hire similarly skilled clinicians but had “deeper pockets” and offer better compensation than community based mental health organizations. Participants also talked about competing with other human service organizations (e.g. child welfare agencies, housing service organizations) that draw from the same workforce:

Finding a clinician to do the work is now becoming quite a problem…. It’s not just mental health, it’s the same licensed people who provide mental health services are the same people that human services are looking for. Children’s services and social services and they’re all trying to pick from the same kind of smaller slice of the pie. (leader from a medium-sized non-profit organization)

Finally, a few leaders even described how they compete for personnel with organizations outside of health and human services including “the Walmarts and the Kohls and the local restaurants that pay about the same.”

Competition for personnel is not restricted to front-line clinicians and program staff—leaders reported strong competition for mid-level managers and executive leaders as well:

Right now, there is a shortage of certified social workers…finding qualified, certified professionals …And then also competing with one another for qualified staff at other levels too. I think it was only ten years ago where 60% of the executive directors were in their sixties, of non-profit agencies. So, there’s a lot who are going to retire, so finding the leadership talent is something else that we compete for…. that’s probably where we get most frustrated with each other. (CEO of a medium-sized non-profit organization)

Such intensive competition for personnel at every level of the organization was perceived to contribute to substantial fluidity of the staff and leadership.

#### Drivers of Workforce Competition

Participants identified three environmental forces that intensified workforce competition. First, leaders attributed theintensive competition to perceived regional shortages of clinical counselors, psychiatrists, nurse practitioners, and master’s-level licensed social workers (key suppliers). Several participants even asked the interviewers about enrollment in the Master of Social Work programs at their home institution, expressing concern that local social work schools were not graduating enough new workers.^1^ Second, leaders reported that new organizations have entered or expanded their reach in the regional market and are driving demand for clinical professionals at the same time. Directors identified several new for-profit behavioral health organizations (that tend to specialize in substance use treatment) recently established in the region that are hiring licensed clinicians.

Third, leaders hypothesized that other types of organizations and employers (substitutes) recruit clinical and other direct service professionals. With the state Medicaid program shifting to a managed behavioral health care model, the five insurance providers selected to manage services are also hiring skilled and trained clinicians, especially licensed social workers, to coordinate benefits and services. Leaders acknowledged that they also lose direct service professionals to organizations outside of the health and human services systems because the emotionally taxing nature of the work leads to burnout:

So, instead of working with a kid who’s gonna try to bite your head off, it’s easier working at WalMart. So, you have that level of competition now that’s going on, because as the economy gets tighter, the pool of workers gets tighter. And that’s where we become much more competitive. (leader of a large for-profit organization)

Thus, as one leader from a large non-profit summed up, “the pool of available talent is shallow, the needs are great.”

#### Organizational Responses to Workforce Competition

In situations where the number of open positions exceeds the supply of potential talent in the region, mental health professionals can be more selective when choosing an employer. As one leader of a small nonprofit noted, “Somebody with a master’s degree and LSW is golden in this field now.” Considering the range of employment choices available, executive leaders reported on three types of strategies for responding to workforce competition. First, organizations differentiate themselves from others in the region when recruiting new employees. One approach involves offering attractive signing bonuses, high salaries, and benefit packages to attract new employees. While some organizations advertise these benefits in their job announcements, others reported taking a more direct approach by deliberately targeting other organizations’ personnel. One leader described how a newer for-profit substance use treatment facility tried to “poach” their clinicians:

Private providers are trying to send postcards to our staff, telling them they can work various hours. That they’re gonna be paid dramatically higher wages and that they’re going to have better benefits and things like that. So, they’re trying to attract our employees. (leader of a large non-profit organization)

Resource constrained organizations may not be able to offer financial incentives. Instead, these organizations might be able to distinguish themselves in the market by focusing their recruitment on newer professionals and offering desirable training or work experiences (a focusing strategy). For instance, one director of a large non-profit organization described their approach to specializing in professional training for new professionals:

So, a lot of the social service agencies struggle to fill positions because the pay scales aren’t usually as robust. The benefits are not usually as robust. And sometimes the work opportunities are not as attractive. So, we sort of carve out our niche as being something of a training ground for people who come out of school with a bachelor’s degree or a master’s degree.

Several leaders described how they felt as though they could not “win” in a competition for new personnel (especially with healthcare organizations with more robust funding). Instead, they described defensive strategies for retaining existing employees (thus reducing the need to recruit). From these conversations emerged a third type of response—organizations sought to differentiate themselves in terms of positive work climate and employee satisfaction. The leaders described a variety of internal changes that have been made with the aim of promoting a supportive workplace climate, improving schedule flexibility, and providing emotional support, given the demanding nature of clinical work. For instance, one leader described how she focused on building a supportive climate by demonstrating her appreciation for staff:

The culture here is much different than other places. And we can’t compete with other agencies for insurance or salaries. But the staff do feel valued and appreciated. And there’s a camaraderie between them. (leader of a medium-sized nonprofit organization)

Generally, these internal management approaches were intended to appeal to and retain clinicians. Leaders believed that clinicians’ decisions to accept an employment offer or stay in their current positions are motivated by non-economic factors like workplace support, flexibility, and appreciation. One leader of a small for-profit organization explained, “we do a lot of nice things for our employees that corporate agencies can’t really do” suggesting that some believe by offering supportive cultures, organizations with limited financial resources motivate experienced staff to resist lucrative recruitment offers from other organizations.

### Competition for Funding/Clients: Historically Weak but Expected to Change

Most leaders reported how they experienced minimal competition for funding (especially through the state’s Medicaid program). As one CEO of a large nonprofit explained:

Historically, I would say for the last, I don’t know, ten, fifteen, twenty years, the competition has been relatively minimal. And the reason is how we’re funded… With Medicaid, we can bring on as many clients as we want, because Medicaid pays the bills. So there really isn’t a competition.

Although several leaders acknowledged that Medicaid and other funders fail to cover the full costs of service delivery, organizations found ways to enhance their efficiency and “make that up in volume.”

#### Historical Drivers of Competition for Funding/Clients

These historically low levels of competition were also attributed to a convergence of environmental factors and early regional system planning efforts. First, leaders described a strong demand for behavioral health services driven by the opioid epidemic in the state. Second, behavioral health services were perceived to be more accessible to the community because of a series of federal and state healthcare policy developments. Specifically, the Affordable Care Act, state Medicaid expansion, and federal behavioral health parity legislation expanded insurance coverage for behavioral health services. Rising community needs and insurance coverage generated strong service demands and reduced direct rivalry among organizations for clients (and by extension, funding).

Third, leaders noted limited direct rivalry for clients because of previous efforts to segment the client population by geography or type of treatment need (a focusing strategy). Among the larger community mental health organizations, leaders described how mental health service planning efforts in the 1970s carved out service areas within the region and designated groups of organizations to deliver care. In the other community-based organizations (that deliver mental health care as part of an array of other human services) leaders described how they carve out a treatment niche that is distinct from other organizations, which reduces competition:

I would say about 15 years ago is when I saw competition being really, really stiff among all social service providers. And in the last five or six, I have seen a steady decline in most of our agencies feelings like we compete. In fact, a lot of us are working much more collaboratively, because a lot of agencies started with specialized and special populations. … Most of us don’t really have to compete because there’s so much demand. So now, over the last five years, competition, is the least of our agency concerns. Because there’s enough business to go around and a lot of us can’t even keep up. (leader of a small for-profit organization)

In some of the smaller mental health organizations, several leaders described their organizations as “boutique” with specialized expertise in delivering mental health care to a specific population (e.g., individuals with developmental disabilities, children with problem sexual behaviors).

The combination of heightened community needs, expanded insurance coverage for behavioral health care, and historic market segmentation resulted in a somewhat flush market for Medicaid-eligible clients. In fact, one leader of a small for-profit organization described the market as a “free for all” and the “wild, wild, West,” where “clients and the people [are viewed] as commodities.” However, leaders in small organizations acknowledged competition for other sources of funding (e.g., grants, child welfare contracts, foundation funding).

#### Transition to Managed Care and Anticipatory Responses

Leaders anticipated that competition (for funding, clients and personnel) would increase dramatically when the state Medicaid program transitioned from a fee-for-service to managed care model that was intended to contain costs by improving care coordination and quality. Under managed care, organizations anticipated that they would compete head-to-head with other organizations for contracts (and thus, revenue) from five private insurance organizations. As leaders understood it, organizations would have a competitive advantage if they could provide an array of coordinated services in-house (enhancing efficiency) and demonstrate improved client outcomes (enhancing quality). Leaders perceived that a managed care model would reduce financial resources available for behavioral health care and direct them toward large organizations (which tended to be connected to major hospital systems or were for-profit entities) that delivered a full range of health and behavioral health services, and had sophisticated infrastructure for evaluating quality/outcomes.

In anticipation of this change, leaders reported two approaches to position their organizations to secure funding in this new environment. First, leaders described continued efforts to expand their programming by launching new services, and/or partnering with other organizations to increase referral volume and coordinate services. Several also described how their organization merged or considered a merger to expand capacity. By growing their programming and client volume, leaders hoped to improve the efficiency of their services. Second, leaders described strategies to improve outcome measurement and monitoring to demonstrate and differentiate themselves among others in terms of service quality. A few also reported adopting evidence-based practices (EBPs), or working toward having their programs designated as EBPs. Leaders acknowledged that these changes were difficult to finance, especially for smaller organizations. As one leader of a small nonprofit explained,

You know, when there’s anything that is a startup, it’s gonna take time to build your revenue and to cover your costs and to eventually be on the positive side of it. But for smaller organizations like ours, I think the risk level is a little bit higher … when you’re smaller, and you don’t have as many revenue streams that you can pull from, you maybe don’t have those same opportunities to support starting up something new, that a bigger organization might have. Because they do have a more diverse funding stream. (leader of a small non-profit organization)

Regardless of size, leaders expressed extreme uncertainty about the impeding shift toward managed care. In fact, several expressed fear about the financial viability of their organization, and others in the region predicted that this shift may lead to consolidation within the regional mental health system. Leaders worried that the large multi-service organizations will thrive under managed care, while the majority of the community-based mental health organizations in the region (which carved out their service niches over the years) will not be competitive and may not survive. An executive director from a large non-profit organization shared,

I predict actually Alicia that community mental health centers like ours will not exist one day. Probably in the next five to ten years, I don’t think an organization like ours what I would call a niche provider will exist in its present form. I think there will be behavioral health services, … But I think they’ll be a component of a larger organization. Cause I’m not sure we can make it financially on our own a whole lot longer…I think we’re running scared, to be honest with you. I know some providers who feel like they’re probably going to be out of business in the first year of behavioral health redesign full implementation. I don’t really know. I honestly can’t tell you. But I certainly know there’s a level of trepidation that I’ve never seen before… So that’s what competition does, it scares you to death, frankly.

### Competitive vs. Collaborative Values in Mental Health Service Delivery

Throughout the interviews, leaders also shared their views about the general impact of competition on mental health service delivery. A minority of these leaders acknowledged that moderate levels of competition can promote accountability:

I think that there’s a lot of really positive things that will come out with this level of accountability. Because then the competition can shift to: we’re going to provide the absolute best service and we’re going to have the data to prove that. That would be, healthy, good competition. That’s the goal … rather than we have to be the biggest and most profitable agency. (Leader of a medium-sized non-profit organization)

By driving organizations to create more supportive workplaces that retain talented employees, or adopt effective interventions, these leaders perceived that competition heightens pressure to demonstrate accountability and can improve service quality throughout the regional system.

However, many leaders felt differently about the value of competition in mental health care, and explained how excessive competition in the field splits limited resources, and negatively impacts service quality:

I think that what it does is it drains resources, it splits resources, and that makes it difficult for any organization to stand in a kind of solid, stable, sustainable, healthy way. Because there’s so much competition. I think that competition then equates to or rolls over into how services are delivered. So, if you are competing for such a small pool of resources, and … you have a case manager who has 150 people on their case load now, that’s not good service. I actually think that it hurts service if you have too much competition for too little resources. (CEO of a medium-sized non-profit organization)

Other leaders described competition as “a business concept, which does not belong in the human service field because we deal with individuals and our bottom line is people’s lives” rather than profits, and another leader called it “wasted energy.”

Leaders described how they frequently partner with organizations that compete for similar resources. Collaborating with a competitor was perceived to confer organizational benefits because organizations can pool their information about the latest community needs, identify service gaps, and plan strategically how each organization will fill them (while preserving service niches). A director of a small nonprofit described how she and her partner “co-mingle resources… and feed off of each other… it’s basically for efficiency purposes.” In addition, leaders believed that collaboration with a competitor improved the quality and coordination of services throughout the region. Smaller organizations that described themselves as “boutique” providers offering a specialized service may be unable to meet all client needs; however, by linking with other similar (competing) organizations they can provide comprehensive services. Several directors believed that partnering with their competitors will lead them to be more efficient and effective, and thus, collectively they will be more competitive for managed care contracts, improving their odds of surviving.

## Discussion

Competition among mental health organizations is poorly understood. In this study, Porter’s competitive forces framework was a useful approach for understanding how organizational leaders manage competition and generating new insights about how mental health policies may be viewed and enacted in the field. We found that the intensity, drivers, and response to competition varies depending on the resource type. Intensive competition for qualified mental health professionals was driven by the vertical pressure of workforce shortages, as well as the horizontal threats of new organizations and employment opportunities outside of the system; these pressures directly influenced organizations’ employee recruitment and retention approaches. On the other hand, competition for clients and funding was minimal because of vertical forces (strong service demand, increased insurance coverage) and a history of strategic efforts to segment the market which eased horizontal pressure. These results suggest that the efficiency and effectiveness objectives of privatization may not have been realized because of low levels of competition for funding and clients. Shifts in Medicaid financing were expected to intensify competition although directors expressed uncertainty, fear, and concern that intensive competition is inconsistent with core values of community-based mental health care. These results have direct implications for policy and management and highlights several important directions for future research.

### Intense Competition for Professional Talent

Organizations competed intensively for qualified personnel, a theme echoed in other research on human service organizations (Collins-Camargo et al. 2019). Behavioral health workforce shortages (a vertical pressure), strong horizontal pressure from new entrants to the market (e.g., for-profit substance use treatment organizations), and employment options in other fields which function as substitutes intensified competition for professional talent. According to Porter, professionals have substantial power under these conditions and can drive up compensation. Indeed, some organizations responded in turn by differentiating themselves in terms of wages and benefits. However, many mental health organizations may struggle to afford higher wages considering limited assets, and low reimbursement rates, especially from Medicaid for services delivered by social workers (who deliver the majority of mental health services) (Bunger et al. 2019; Watson et al. 2013). Instead of addressing the seemingly intractable challenge of salaries, some leaders are defining this challenge in ways that might be more under their control, such as work-life balance, organizational culture and professional development (defensive tactics to retain current staff). This is a common approach in knowledge-intensive fields (von Nordenflycht 2010) and seems wise given the difficulty of raising salaries and considering how factors other than salary often drive clinicians to leave, or intent to leave a current employer (Acker 2004; Fukui et al. 2019).

Behavioral health workforce shortages have been well-documented and are projected to continue given service demands, and current training capacity (Covino 2019; Hoge et al. 2013; McBain et al. 2019; Satiani et al. 2018). Under continued or escalating competition for personnel, mental health organizations may struggle to maintain a qualified workforce which could have adverse effects for service quality (turnover, supervision, etc.). Organizations could address undersupply by reducing minimal degree and credentialing standards, or recruiting other types of professionals (e.g., lay health workers, peer support) (Hoge et al. 2013). However, none of the leaders in this study reported using this strategy. This may be driven by an organizational commitment to preserve high quality service delivery or governmental and insurance reimbursement rates (preferencing doctorate-level providers). Regardless, training incentives and apprenticeship programs that offer new clinicians clear pathways to the local workforce will continue to be important (Hoge et al. 2013).

### Limited Competition for Funding and Clients: The Role of Policy

The leaders in this sample reported that historically, competition for funding and clients has been minimal. Drawing on Porter’s framework, this weak competition can be explained partially by a history of strategic responses (e.g., carving out service niches) to reduce horizontal competition from other local organizations. For instance, the organizations in this sample represent a diversity of facility types, sizes, and target populations which could reduce the degree to which they compete with one another directly. However, surges in service demand (e.g., opioid crisis) coupled with expanded insurance coverage reduced vertical pressures exerted by client and funders’ “buying power.” Thus, there have been few competitive pressures to intensify organizational rivalry over the past several decades despite a general push toward privatization. This implies that federal, state, and local responses have not been consistent in their aim of promoting and leveraging competition in the private market.

Shifting to a managed care model for administering behavioral health services through the state’s Medicaid program could intensify competition, as leaders in this study anticipated. Managed care models are indeed intended to stimulate competition—but among insurance carriers. Under managed care, selected insurance plans compete for Medicaid-enrollees, who theoretically choose a carrier based on the quality of their provider networks (cost is not a factor in the decision since Medicaid-enrollees do not pay premiums) (Enthoven 1993; Frank and Glied 2006). The managed care entities are incentivized to keep service costs down by establishing a manageable network of providers that deliver effective services efficiently, and in coordination with others in the network (McGuire 2016). Thus, they might only offer a limited number of contracts, preferencing larger multi-service organizations. In this sample, leaders expected that they will have to compete intensively for these contracts in order to continue serving (and be reimbursed for serving) Medicaid-enrollees. These findings suggest that Medicaid managed care models (which increase state Medicaid programs’ buying power, a vertical pressure) might also stimulate downstream competition for funding among local organizations. It also suggests that clients’ choice (and thus their power as service consumers) might also be restricted as they are directed to services by their managed care coordinator which may have negative consequences for organizational responsiveness to consumer needs (McBeath et al. 2014).

Losing a Medicaid contract could threaten the viability of these organizations, since many depended on Medicaid-enrollees as their primary clientele. As reported in this study, efforts to expand services through mergers, collaboration, or other growth (perhaps to counter the specialized niches developed over the years) and implement evidence-based practices were intended to make organizations more attractive as managed care network members. These organizational responses (growth, mergers, and attention to quality) have potential to improve services as observed in the general healthcare sector (Postma and Roos 2016), but must be examined in future studies.

### Potential Consequences of Competition for Mental Health Service Organizations

While competition (or the anticipation of competition) might trigger strategic responses to improve work conditions and service quality, our study also points to the potential downsides of competition. Leaders feared that intensive competition for funding and clients could starve organizations of resources needed for recruiting talented personnel, improving quality, and innovation. Some predicted that small or mid-sized organizations (those that do not win managed care contracts) would merge or fold. Although it could be argued that dissolution is a natural consequence for ineffective or inefficient organizations, a contraction in the size and scope of regional mental health systems could negatively affect service diversity and availability. As voiced by leaders in this study, these potential consequences raise questions about whether competition is consistent with the mission and values of human service delivery.

Although leaders’ opinions about the value of competition was not our original study intent, the participants’ views in this study point to several new insights about mental health policies. Policy and other environmental changes that alter competition among organizations can lead to substantial uncertainty for leaders and difficulty predicting outcomes of their decisions (Milliken 1987). So that leaders and their boards can prepare their organizations, funders and policy makers should communicate expectations and timelines clearly for planned policy shifts that intensify competition. Our findings also suggest that small and mid-sized organizations might need additional assistance (e.g., time, funding, technical support around billing) so that they can compete with larger, or better-resourced organizations. Notably, leaders in this sample felt strongly that they are more effective when they collaborate than compete, in line with prior studies demonstrating a strong relationship between collaboration and a desire to meet emergent community needs (Bunger et al. 2017; Selden et al. 2006). Consistent with this belief, they described collaborative strategies to expand in preparation for a more competitive environment (e.g., via referral partnerships, alliances, mergers), suggesting that they would be more competitive as a small group than an individual organization. These results help explain why mental health and other human service organizations frequently collaborate with their direct competitors (Bunger 2013; Bunger et al. 2014) and implies that competition might emerge between small groups rather than among individual organizations (Bunger and Gillespie 2014; Trapido 2007), although this hypothesis should be tested in future studies. As an alternative to policy and system reforms that promote competition, policy makers and funders might support new organizational alliances among small groups of organizations, especially considering how systems organized around highly collaborative organizational groups have been associated with client outcomes (Lemieux-Charles et al. 2005; Provan et al. 1996).

### Who Really Benefits from Competition Among Mental Health Organizations?

Theoretically, market competition is presumed to benefit consumers by driving innovation, effectiveness, and cost-efficiency. Although we do not test this proposition, our study provides minimal support for the idea that competition directly benefits consumers, at least in this sample of organizations. Leaders reported a few efforts to improve treatment quality or consumer experience (e.g. implement EBPs) however, these were rare; we heard little about how organizations are reducing out of pocket costs, enhancing accessibility, or responding to issues of equity, for example. Instead, leaders responded to competition (current and anticipated) by attending to workplace culture, salaries, service volume, etc. Thus, the principal beneficiaries of competition among these mental health organizations appeared to be funders and the workforce, not the individual client.

For consumers to benefit from competition, they require both a choice of service providers, and the capacity to make an informed judgement about service quality. In this study, providers’ historical efforts to segment the market geographically and by service type likely limited clients’ choice. Additionally, defining quality of care in mental health treatment is a complex and emerging construct (Kilbourne et al. 2018)—one that far exceeds the capacity of most consumers even if there are clear outcome indicators and data available. While there is some support for a relationship between client satisfaction and quality of care in mental health (Edlund et al. 2003), there is no mechanism for communicating that information to consumers when they are seeking services, beyond perhaps word of mouth or online reviews.

### Limitations and Directions for Future Research

Our findings should be considered in light of limitations. Our results were influenced by the context of the sample setting, which included impending Medicaid shifts and professional licensure requirements in the state, combined with regional behavioral health needs (particularly in the wake of an opioid crisis) and available qualified professionals. The sample also primarily consisted of nonprofit organizations; while the interviews we conducted with for-profit leaders did not generate unique insights, we recommend a targeted exploration of whether leaders experience competition differently in a for-profit environment. In addition, other stakeholders (e.g., staff, funders, consumer) might have different perspectives on competition and how organizations respond which are not reflected in this study. However, we took several measures to enhance the validity of results; three team members coded the interviews and met several times to refine the codebook and reach consensus on applied codes. Although we reached saturation of themes within our small sample of interviews, other competitive pressures might be identified in other regional or policy contexts.

This qualitative study was conducted to expand our limited understanding of competition among mental health organizations and our findings point to several implications for a continuing line of research. First, our study points to the need for a more robust measurement of competition in mental health markets. Traditionally, competition has been captured using the Herfindahl–Hirschman index, a quantitative metric of market concentration that reflects the number of organizations and market size (total revenues) (Rhoades 1993). However, as Porter’s framework and our study illustrates, this may offer a full depiction of the competitive environment. A quantitative measure might reflect the perceived intensity of competition for resources at the organizational level (e.g. funding, contracts, staff), or the salience/intensity of Porter’s five forces at the market level.

Second, our findings related to how administrative and financing reorganizations (e.g. parity, Medicaid expansion, shift to managed care) influence competition, and organizational change suggests the need to examine interorganizational competition as a potential mediator in policy studies. For instance, studies examining the impact of managed behavioral health care might examine intensity of competition for managed care contracts, the preferred features of organizations that win contracts, and the effects on care availability, quality, costs, and the population of community-based behavioral health organizations. This could be examined in a multi-level study that links data on the number and service scope of mental health organizations in a region, interorganizational competition for managed care contracts, characteristics of organizations awarded a contract, and claims data. Expanding research on competition into other geographic regions, and over time would likely reveal new insights into behavioral health markets depending on varying political, community, and workforce factors.

Third, our findings identify several ways organizations respond to changing competitive conditions (e.g. implementing EBPs, improving culture, sign-on bonuses), the effectiveness of which are likely to be of interest to organizational leaders and funders alike. Studies that test the system and organizational conditions under which these strategies are most useful could inform managers as they respond to varying market forces. Importantly, these studies should examine the impact on client outcomes (especially outcomes deemed most relevant and important to clients) to determine whether and how organizational response to competition benefit consumers. Finally, since our study suggests that system reform efforts to increase competition may not have achieved their intended goals, there may also be value in understanding funders’ decision-making processes. This might include exploring how funders balance cost containment with quality, operationalize quality, fund quality assessments, and hold organizations accountable.

## Conclusions

Mental health system reforms have sought to leverage competition (particularly for funding and clients) in the private sector to improve service quality and costs, despite an incomplete understanding of how mental health organizations experience and respond to competition. Our study demonstrates that mental health organizations do compete—for qualified personnel—and they adjust in ways that might improve services, consistent with theory on competition and the underlying goals of privatization. However, our study also suggests that privatization’s promised performance gains may remain unrealized because of minimal competition for funding and clients (driven by a history of segmenting the local market, federal policies expanding treatment access, and strong community need). Although the shift to managed care model might intensify competition, our participants also questioned whether such intensive competition is consistent with the values of community-based service delivery. As our field considers and evaluates alternative models for structuring and financing mental health services, the intensity, drivers, and organizational responses to the competitive environment are likely to be salient explanations for the impacts observed.

## Footnotes

1 Records from the largest MSW program in the region indicate that MSW enrollment was at an all-time high.

## References

[CR1] Acker GM (2004). The effect of organizational conditions (role conflict, role ambiguity, opportunities for professional development, and social support) on job satisfaction and intention to leave among social workers in mental health care. Community Mental Health Journal.

[CR2] Alexander JA, Wells R, Jiang L, Pollack H (2008). Organizational determinants of boundary spanning activity in outpatient substance abuse treatment programmes. Health Services Management Research : An Official Journal of the Association of University Programs in Health Administration/HSMC, AUPHA.

[CR3] Apenteng BA, Nayar P, Yu F, Adams J, Opoku ST (2015). Organizational and environmental correlates of the adoption of a focus strategy in U.S. hospices. Health Care Management Review.

[CR4] Baker WE, Faulkner RR, Fisher GA (1998). Hazards of the market: The continuity and dissolution of interorganizational market relationships. American Sociological Review.

[CR5] Barman EA (2002). Asserting difference: The strategic response of nonprofit organizations to competition. Social Forces.

[CR6] Barnett WP, Greve HR, Park DY (1994). An evolutionary model of organizational performance. Strategic Management Journal.

[CR7] Baum JAC, Singh JV (1994). Organizational niches and the dynamics of organizational mortality. American Journal of Sociology.

[CR8] Berrone P, Gelabert L, Massa-Saluzzo F, Rousseau HE (2016). Understanding community dynamics in the study of grand challenges: How nonprofits, institutional actors, and the community fabric interact to influence income inequality. Academy of Management Journal.

[CR9] Boehm A (1996). Forces driving competition in human service organizations and positional competitive responses. Administration in Social Work.

[CR10] Brandenburger A, Nalebuff BJ (1996). Co-opetition.

[CR11] Breedveld EJ, Meijboom BR, de Roo AA (2006). Labour supply in the home care industry: A case study in a Dutch region. Health Policy (Amsterdam, Netherlands).

[CR12] Brunjes BM (2019). Competition and federal contractor performance. Journal of Public Administration Research and Theory.

[CR13] Bruns EJ, Kerns SEU, Pullmann MD, Hensley SW, Lutterman T, Hoagwood KE (2016). Research, data, and evidence-based treatment use in state behavioral health systems, 2001–2012. Psychiatric Services.

[CR14] Bunger AC (2013). administrative coordination in nonprofit human service delivery networks: The role of competition and trust. Nonprofit and Voluntary Sector Quarterly.

[CR17] Bunger AC, Gillespie DF (2014). Coordinating nonprofit children’s behavioral health services: Clique composition and relationships. Health Care Management Review.

[CR15] Bunger AC, Collins-Camargo C, McBeath B, Chuang E, Pérez-Jolles M, Wells R (2014). Collaboration, competition, and co-opetition: Interorganizational dynamics between private child welfare agencies and child serving sectors. Children and Youth Services Review.

[CR16] Bunger AC, Despard M, Lee M, Cao Y (2019). The cost of quality: Organizational financial health and program quality. Journal of Evidence-Informed Social Work.

[CR18] Bunger AC, McBeath B, Chuang E, Collins-Camargo C (2017). Institutional and market pressures on interorganizational collaboration and competition among private human service organizations. Human Service Organizations: Management, Leadership & Governance.

[CR19] Clark RE, Dorwart RA (1992). Competition and community mental health agencies. Journal of Health Politics, Policy and Law.

[CR20] Collins-Camargo C, Chuang E, McBeath B, Mak S (2019). Staying afloat amidst the tempest: External pressures facing private child and family serving agencies and managerial strategies employed to address them. Human Service Organizations Management, Leadership and Governance.

[CR21] Compagni A, Mele V, Ravasi D (2014). How early implementations influence later adoptions of innovation: Social positioning and skill reproduction in the diffusion of robotic surgery. Academy of Management Journal.

[CR22] Covino NA (2019). Developing the behavioral health workforce: Lessons from the states. Administration and Policy in Mental Health and Mental Health Services Research.

[CR23] Cuellar AE, Haas-Wilson D (2009). Competition and the mental health system. The American Journal of Psychiatry.

[CR24] Dess GG, Davis PS (1984). Porter’s (1980) generic strategies as determinants of strategic group membership and organizational performance. Academy of Management Journal.

[CR25] Domański J (2012). Competitiveness of nongovernmental organizations in developing countries: Evidence from Poland. Nonprofit and Voluntary Sector Quarterly.

[CR26] Edlund MJ, Young AS, Kung FY, Sherbourne CD, Wells KB (2003). Does satisfaction reflect the technical quality of mental health care?. Health Services Research.

[CR27] Eikenberry AM, Kluver JD (2004). The marketization of the nonprofit sector: Civil society at risk?. Public Administration Review.

[CR28] Enthoven AC (1993). The history and principles of managed competition. Health Affairs.

[CR29] Frank RG, Glied SA (2006). Better but not Well: Mental Health Policy in the United States Since 1950.

[CR30] Fukui S, Wu W, Salyers MP (2019). Impact of supervisory support on turnover intention: The mediating role of burnout and job satisfaction in a longitudinal study. Administration and Policy in Mental Health and Mental Health Services Research.

[CR31] Gaynor M, Ho K, Town RJ (2015). The industrial organization of health-care markets. Journal of Economic Literature.

[CR32] Girth AM, Hefetz A, Johnston JM, Warner ME (2012). Outsourcing public service delivery: Management responses in noncompetive markets. Public Administration Review.

[CR33] Gnyawali DR, Park B-J (2009). Co-opetition and technological innovation in small and medium-sized enterprises: A multilevel conceptual model. Journal of Small Business Management.

[CR34] Gray BH, Schlesinger M, Salamon LM (2002). Health. The state of nonprofit America.

[CR35] Grønbjerg KA (1993). Understanding nonprofit funding : Managing revenues in social services and community development organizations.

[CR36] Guo C, Acar M (2005). Understanding collaboration among nonprofit organizations: Combining resource dependency, institutional, and network perspectives. Nonprofit and Voluntary Sector Quarterly.

[CR37] Hogan MF (2003). The president’s new freedom commission: Recommendations to transform mental health care in America. Psychiatric Services.

[CR38] Hoge MA, Stuart GW, Morris J, Flaherty MT, Paris M, Goplerud E (2013). Mental Health and addiction workforce development: Federal leadership is needed to address the growing crisis. Health Affairs.

[CR39] Hu Q, Huang K, Chen B (2019). Professional friendship, resource competition, and collaboration in a homeless service delivery network. Human Service Organizations Management, Leadership and Governance..

[CR40] Hunt SD (1997). Competing through relationships: Grounding relationship marketing in resource-advantage theory. Journal of Marketing Management.

[CR41] Jaskyte K (2013). Does size really matter? Organizational size and innovations in nonprofit organizations. Nonprofit Management and Leadership.

[CR42] Johnston JM, Girth AM (2012). Government contracts and “managing the market”: Exploring the costs of strategic management responses to weak vendor competition. Administration & Society.

[CR43] Kilbourne AM, Beck K, Spaeth-Rublee B, Ramanuj P, O’Brien RW, Tomoyasu N, Pincus HA (2018). Measuring and improving the quality of mental health care: A global perspective. World Psychiatry.

[CR44] Lamothe S (2014). How Competitive is “competitive” procurement in the social services?. The American Review of Public Administration.

[CR45] Lemieux-Charles L, Chambers LW, Cockerill R, Jaglal S, Brazil K, Cohen C (2005). Evaluating the effectiveness of community-based dementia care networks: The Dementia Care Networks’ Study. The Gerontologist.

[CR46] Lethbridge J (2011). Understanding multinational companies in public health systems, using a competitive advantage framework. Globalization and Health.

[CR47] McBain RK, Kofner A, Stein BD, Cantor JH, Vogt WB, Yu H (2019). Growth and distribution of child psychiatrists in the United States: 2007–2016. Pediatrics.

[CR48] McBeath B, Collins-Camargo C, Chuang E (2012). The role of the private sector in child welfare: Historical reflections and a contemporary snapshot based on the national survey of private child and family serving agencies. Journal of Public Child Welfare.

[CR49] McBeath B, Jolles MP, Chuang E, Bunger AC, Collins-Camargo C (2014). Organizational responsiveness to children and families: Findings from a national survey of nonprofit child welfare agencies. Children and Youth Services Review.

[CR50] McGuire TG (2016). Achieving mental health care parity might require changes in payments and competition. Health Affairs.

[CR51] Milliken FJ (1987). Three types of perceived uncertainty about the environment: State, effect, and response uncertainty. The Academy of Management Review.

[CR52] Milward HB, Provan KG (2000). Governing the hollow state. Journal of Public Administration Research and Theory.

[CR53] Owens PL, Hoagwood K, Horwitz SM, Leaf PJ, Poduska JM, Kellam SG, Ialongo NS (2002). Barriers to children’s mental health services. Journal of the American Academy of Child & Adolescent Psychiatry.

[CR81] Porter ME (1980). Competitive strategy.

[CR54] Porter ME (2008). On competition.

[CR55] Porter ME (2008). The five competitive forces that shape strategy. Harvard Business Review.

[CR56] Porter ME, Teisberg EO (2006). Redefining health care: Creating value-based competition on results.

[CR57] Postma J, Roos A-F (2016). Why healthcare providers merge. Health Economics Policy and Law.

[CR58] Proctor EK, Knudsen KJ, Fedoravicius N, Hovmand P, Rosen A, Perron B (2007). Implementation of evidence-based practice in community behavioral health: Agency director perspectives. Administration and Policy in Mental Health and Mental Health Services Research.

[CR59] Provan KG, Sebastian JG, Milward HB (1996). Interorganizational cooperation in community mental health: A resource-based explanation of referrals and case coordination. Medical Care Research and Review.

[CR60] Reid GJ, Brown JB (2008). Money, case complexity, and wait lists: Perspectives on problems and solutions at children’s mental health centers in Ontario. The Journal of Behavioral Health Services & Research.

[CR61] Rhoades SA (1993). The Herfindahl-Hirschman Index. Federal Reserve Bulletin.

[CR62] Romzek BS, LeRoux K, Blackmar JM (2012). A preliminary theory of informal accountability among network organizational actors. Public Administration Review.

[CR63] Sandfort JR (2003). Exploring the structuration of technology Within Human Service Organizations. Administration & Society.

[CR64] Satiani A, Niedermier J, Satiani B, Svendsen DP (2018). Projected workforce of psychiatrists in the United States: A population analysis. Psychiatric Services.

[CR65] Savas ES (2002). Competition and choice in New York City. Social Services. Public Administration Review.

[CR66] Schensul SL, Schensul JJ, LeCompte MD (1999). Essential ethnographic methods: Observations, interviews and questionnaires.

[CR67] Selden SC, Sowa JE, Sandfort J (2006). The impact of nonprofit collaboration in early child care and education on management and program outcomes. Public Administration Review.

[CR80] Shulman, S. W. (2017).* Coding analysis toolkit*. Texifter. [online] URL: http://cat.texifter.com/.

[CR68] Smircich L, Stubbart C (1985). Strategic management in an enacted world. The Academy of Management Review.

[CR69] Smith SR, Smyth J (1996). Contracting for services in a decentralized system. Journal of Public Administration Research and Theory.

[CR70] Strauss A, Corbin J (1998). Basics of qualitative research: Techniques and procedures for developing grounded theory.

[CR71] Trapido D (2007). Competitive embeddedness and the emergence of interfirm cooperation. Social Forces.

[CR72] Tuckman HP (1998). Competition, commercialization, and the evolution of nonprofit organizational structures. Journal of Policy Analysis and Management.

[CR73] Valente TW, Coronges KA, Stevens GD, Cousineau MR (2008). Collaboration and competition in a children’s health initiative coalition: A network analysis. Evaluation and Program Planning.

[CR74] Van Slyke DM (2003). The mythology of privatization in contracting for social services. Public Administration Review.

[CR75] Vangen S, Huxham C (2003). Enacting leadership for collaborative advantage: Dilemmas of ideology and pragmatism in the activities of partnership managers. British Journal of Management.

[CR76] von Nordenflycht A (2010). What is a professional service firm? Toward a theory and taxonomy of knowledge-intensive firms. Academy of Management Review.

[CR77] Watson LD, Milam SP, Cooper CB, Hansen V (2013). Medicaid reimbursement policy for mental health services in Texas: Impact on service access and level of LCSW social workers participation in medicaid. Journal of Policy Practice.

[CR78] Westra D, Wilbers G, Angeli F (2016). Stuck in the middle? A perspective on ongoing pro-competitive reforms in Dutch mental health care. Health Policy.

[CR79] Xu WY, Song C, Li Y, Retchin SM (2019). Cost-sharing disparities for out-of-network care for adults with behavioral health conditions. JAMA Network Open.

